# Deep neural network modeling for brain tumor classification using magnetic resonance spectroscopic imaging

**DOI:** 10.1371/journal.pdig.0000784

**Published:** 2025-04-09

**Authors:** Erin B. Bjørkeli, Knut Johannessen, Jonn Terje Geitung, Anna Karlberg, Live Eikenes, Morteza Esmaeili

**Affiliations:** 1 Department of Diagnostic Imaging, Akershus University Hospital, Lørenskog, Norway; 2 Institute of Clinical Medicine, University of Oslo, Oslo, Norway; 3 Department of Circulation and Medical Imaging, Norwegian University of Science and Technology (NTNU), Trondheim, Norway; 4 Department of Radiology and Nuclear Medicine, St. Olavs Hospital, Trondheim University Hospital, Trondheim, Norway; 5 Department of Electrical Engineering and Computer Science, University of Stavanger, Stavanger, Norway; University of Cambridge, UNITED KINGDOM OF GREAT BRITAIN AND NORTHERN IRELAND

## Abstract

This study is driven by the complex and specialized nature of magnetic resonance spectroscopy imaging (MRSI) data processing, particularly within the scope of brain tumor assessments. Traditional methods often involve intricate manual procedures that demand considerable expertise. In response, we investigate the application of deep neural networks directly to raw MRSI data in the time domain. Given the significant health risks associated with brain tumors, the necessity for early and accurate detection is crucial for effective treatment. While conventional MRI techniques encounter limitations in the rapid and precise spatial evaluation of diffuse gliomas, both accuracy and efficiency are often compromised. MRSI presents a promising alternative by providing detailed insights into tissue chemical composition and metabolic changes. Our proposed model, which utilizes deep neural networks, is specifically designed for the analysis and classification of spectral time series data. Trained on a dataset that includes both synthetic and real MRSI data from brain tumor patients, the model aims to distinguish MRSI voxels that indicate pathological conditions from healthy ones. Our findings demonstrate the model’s robustness in classifying glioma-related MRSI voxels from those of healthy tissue, achieving an area under the receiver operating characteristic curve of 0.95. Overall, these results highlight the potential of deep learning approaches to harness raw MR data for clinical applications, signaling a transformative impact on diagnostic and prognostic assessments in brain tumor examinations. Ongoing research is focused on validating these approaches across larger datasets, to establish standardized guidelines and enhance their clinical utility.

## Author summary

MRI is the gold standard method for diagnosing brain tumors, determining their type and location, as well as for planning surgery and treatment. Interestingly, the same MRI scanner can also provide metabolic information about the tumor tissue. This technique, called MRSI (Magnetic Resonance Spectroscopic Imaging), gives detailed information about tissue metabolism in different parts of the brain, which is very helpful for diagnosis and early treatment assessment. However, the current methods for processing MRSI data are time-consuming and require specialized hardware and software, which limits the use of MRSI in clinical settings. This study shows that raw MR spectra, without any preprocessing, can effectively be used to classify tumors and healthy brain tissue. This approach eliminates the need for additional preprocessing steps, making it easier to use this advanced diagnostic technique in everyday medical practice.

## 1 Introduction

Brain tumors present a significant public health concern, necessitating early detection and classification for effective treatment [1]. While conventional magnetic resonance imaging (MRI) is widely employed for this purpose, it faces limitations in accuracy and efficiency [2,3]. Magnetic resonance spectroscopic imaging (MRSI), an advanced technique that provides insight into tissue chemical composition, holds promise as a valuable tool in the diagnostic and treatment assessment of gliomas [4–6]. Despite its proven value in research and clinical applications, MRSI encounters challenges in implementation and analysis. Lower signal-to-noise ratio (SNR) during data acquisition affects metabolite quantification accuracy, and the processing and analysis of the data demand specialized expertise [7,8]. The trade-off between spatial and spectral resolution further complicates MRSI. Technological developments aim to address these challenges, yet widespread accessibility to advanced protocols remains limited. Standardized analysis pipelines, software tools, and a unified data format are not entirely optimized; however, there have been concerted efforts, particularly through consortium initiatives [8–10]. Overcoming these challenges holds the potential to significantly enhance the effectiveness of MRSI in brain tumor examination.

Deep learning models, a subset of machine learning algorithms, have had significant success across various medical imaging applications, including brain tumor diagnosis and classification [11,12]. The recent effectiveness of deep learning, particularly convolutional neural networks (CNNs), in MRSI is promising [8,13–18]. These algorithms autonomously learn relevant features from data and thus eliminate the need for manual feature extraction. Deep learning models have been shown to be successful in tasks such as classifying spectra based on clinical assessment quality [15] and distinguishing mutation statuses in gliomas based on MRI images [19]. Deep learning can potentially contribute to the automation of MRSI data analysis, which is expected to save time and make advanced MRSI techniques more accessible to clinics that lack extensive experience with traditional vendor-provided protocols.

Our research addresses the inefficiency in traditional MRSI pipelines due to extensive post-processing. We hypothesize that directly using raw MRSI data—unprocessed time-domain MR signals from the MRI scanner—can characterize tumor-specific features and therefore be used to accurately classify voxels with brain tumors. We propose a deep-learning model to classify tumor-containing voxels from healthy voxels based on spectral time series inputs. The proposed model consists of two distinct blocks. The first step involves training the model on synthetic MR spectra and phantom data to assess its success in domain transformation from raw data to the frequency domain. This first block serves as a foundational step to validate the CNN’s ability to handle spectral data transformations, establishing a baseline performance using synthetic data to be benchmarked against real patient data. The second block classifies the output spectra from the first block as either stemming from glioma or healthy brain tissue. Finally, we trained the entire model on *in vivo* MRSI data to classify gliomas from healthy MR spectra.

## 2 Materials and methods

### 2.1 Simulated brain MR spectra

Dataset simulation followed the approach by Lee and Kim [17], in order to generate ground truth target spectra from a spectral basis set consisting of key brain metabolites. The metabolites included in the basis set were aspartate (Asp), creatine (Cr), gamma-aminobutyric acid (GABA), glutamate (Glu), glutamine (Gln), glutathione (GSH), glycine (Gly), glycerophosphocholine (GPC), glycerophosphoethanolamine (GPE), Myo-inositol (Ins), lactate (Lac), N-acetyl-aspartate (NAA), N-acetyl-aspartyl glutamate (NAAG), phosphocholine (PCh), phosphocreatine (PCr), phosphorylethanolamine (PEtn), scyllo-Inositol (ScyIns), serine (Ser), and taurine (Tau).

For each simulated spectrum, concentrations for each metabolite were randomly selected within the range of metabolite concentrations in normal adult brains according to the literature [17,20,21] based on their intensity in the spectrum. Augmentation was then introduced to the spectra through phase modulation, Lorentzian line broadening, and white noise. These augmented spectra served as training input for the deep learning model. Augmentation served to both make the simulated spectra more realistic and to increase the dataset size to mitigate model overfitting. The white noise, with a magnitude of [0.05 1], aimed to achieve an SNR ranging from 1 to 3, as determined by the ratio of the creatine signal to the white noise magnitude (Table 1). Subsequently, after Fourier transformation, the simulated spectra were sampled at 1024 points and normalized to the range [0,1] to enhance efficiency during model training. A total of 50,000 MR spectra were simulated, with 80% (40,000) allocated for model training, and 10% (5,000) each reserved for validation and testing.

**Table 1:** Parameters used for dataset simulation.

	Lower limit	Upper limit
Zero-order phase shift	-π/4	π/4
Line broadening	0	40 Hz
White noise magnitude	0.05	1
Field strength	3	7 Tesla
Echo time	35 ms	
Metabolite Concentration	Min [mmol/liter]	Max [mmol/liter]
Ala	0.1	1.5
Asp	1.0	2.0
Cr	5.5	7.0
GABA	1.0	2.0
Glc	1.0	2.0
Gln	1.5	5.0
Glu	6.0	9.0
Gly	1.5	2.5
GPC	0.5	2.0
GPE	0.5	2.0
GSH	1.5	3.0
Ins	3.0	5.0
Lac	0.2	1.0
NAA	7.0	10.0
NAAG	0.5	3.0
PCh	0.5	2.0
PCr	3.0	5.5
PEtn	1.0	2.0
Ser	0.5	1.0
ScyIns	0.1	0.3
Tau	2.0	6.0

### 2.2 Brain phantom experiments

An in-house-made structural-metabolic phantom was assessed, featuring six tubes each with a 25 mm diameter, symmetrically positioned within a larger cylindrical container measuring 110 mm in diameter. These six tubes were filled with buffered solutions (pH = 7) of brain metabolites: 6 mM of NAA, 8 mM of glutamate, 1 mM of GABA, 4 mM of creatine, 5 mM of choline, 8 mM of Myo-inositol, and 4 mM of lactate. Each small tube contained slightly different concentrations and combinations of the above-mentioned metabolites. To expedite T1-relaxation, the tube solutions were doped with Magnevist at a concentration of 1 ml/L. Contrarily, the larger cylindrical container was filled with an aqueous solution devoid of metabolites and remained free from Magnevist doping. MRSI data were acquired using a 3 Tesla Philips MRI system (Ingenia, Best, the Netherlands) with the following acquisition parameters: PRESS sequence, TR/TE = 2000/35, matrix size of 32×32, and voxel size of 1×1×5 mm^3^. MR spectra were augmented with phase and intensity modulations, Lorentzian line broadening, and adding white noise, which resulted in a total of 10,000 MR spectra all with 1024 complex data points.

### 2.3 Human subjects

#### Training dataset.

Multi-institutional clinical data from healthy volunteers (n=21) and glioma patients (n=1) including MR spectroscopic data sourced openly from Hingerl et al. [22] and Archibald et al. [23], and from national/international hospital partners were used in the current study. The data spans various magnetic field strengths, including 7 and 3 Tesla, across Philips and Siemens MRI systems, and involves different acquisition sequences. Hingerl L. and colleagues [22] acquired 3-dimensional free induction decay (FID)-MRSI from glioma (n=1) and healthy subjects (n=4) using 7 T (Magnetom, Siemens Healthcare, Erlangen, Germany, maximum gradient strength = 40 mT/m, maximum slew rate = 200 mT/m/ms) with following acquisition parameters: TR/TE=280/1.3 milliseconds; flip angle of 30°, 2778 Hz spectral bandwidth, and one average. Archibald J. and colleagues [23] obtained MRSI data from 15 healthy subjects using a 3 Tesla Philips (Achieva scanner, Best, the Netherlands). The acquisition involved a single-channel Transmit-Receive (T/R) head coil and utilized the Point RESolved Spectroscopy (PRESS) sequence with parameters TR/TE = 4000/22 ms, 32 averages, scan time = 3:12. A total of 16 non-water suppressed spectra were acquired as a baseline, and these spectra were utilized in the current study. Although *in vivo* data already contain noise, we further augmented it by adding Gaussian noise with varying standard deviations or filtering to simulate different levels of signal-to-noise ratios (ranging from 1 to 5, as determined by the ratio of the creatine peak-to-peak signal to the standard deviation of baseline on 8.0-9.0 ppm). Also, MR spectra were augmented with phase and intensity modulations. In total, we prepared about 20,000 augmented *in vivo* MR spectra for training and validation.

#### Test dataset.

MRSI data were also acquired from 40 post-operative patients with glioma (grades 2-4, 20 females, ages 25-74 years) from a national hospital. Anatomical MRI and proton 2D MRSI were acquired using PET/MRI systems (Siemens Biograph mMR, software version Syngo MR, Erlangen, Germany). Standard MRI sequences were acquired, including pre- and post-contrast enhanced (ce) 3D T1 magnetization prepared rapid gradient echo imaging (MPRAGE), 3D FLAIR, and axial T2-weighted imaging. MRSI data parameters included: 2-dimensional PRESS sequence, TR/TE = 1700/30 ms, flip angle of 90˚, three averages, matrix size of 16 × 16, vector size of 1024, and slice thickness of 16 mm.

Ethical approval for the study was granted by the Regional Ethics Committee (REC Central Norway, reference numbers 2016/279 and 2018/2243), aligning with the principles of the Helsinki Declaration and national guidelines. All patients signed written informed consent.

### 2.4 Architecture of neural network

**Fig 1:**
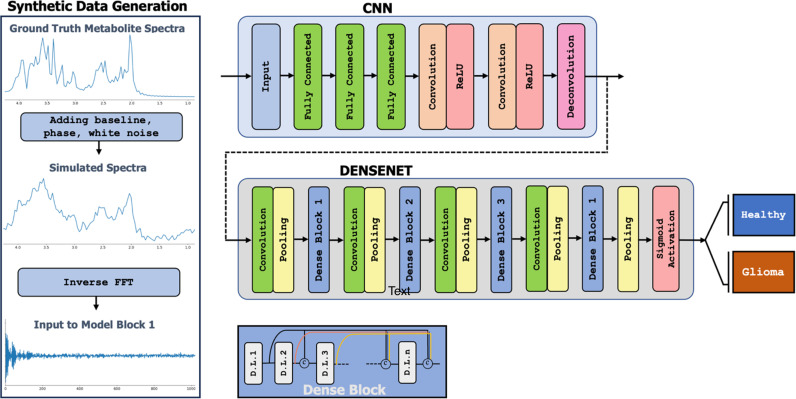
The model architecture and pipeline for generating augmented MR spectra and training the DenseNet-derived model consist of two primary blocks. The first block, “CNN,” is designed for domain transformation and denoising of the input raw spectroscopy data. The second block, “DENSENET,” is intended for classifying the input data. This comprehensive illustration outlines the intricacies of the process, capturing the synthesis, phantom, and clinical MR spectra, and the subsequent training of the DenseNet model.

To build the model, we began by training and evaluating the first block of the model, “CNN”, which was responsible for transforming time-series free induction decay signals into frequency domain MR spectral representations. The primary objective was to ensure that our model could transform time domain complex series into frequency domain MR spectra, extract significant features, denoise the data, and prepare this block as the first stack of the main model. The CNN was trained on a dataset comprising synthetic spectroscopic data and phantom MRS data. For model training and validation, we utilized a dataset of 80,000 MR spectra with 1024 time points, created through a combination of simulated spectra and data from a phantom containing crucial brain metabolites. The ground truth was established using standard spectral processing techniques, including phase modulation and Fourier Transformation in MATLAB (MathWorks, Natick, MA, USA). After successful performance and satisfactory validation, the CNN block was integrated with the second block of the model, “DENSENET” (Fig 1), while preserving the optimized weights. This combined model would be employed to investigate the main goal of this study: classifying raw MRS data from voxels containing glioma versus healthy tissues. We used MRS data acquired from humans to train the model. Notably, the data were not water-suppressed, meaning that the water signal would be significantly larger than that of the metabolites of interest, assuming imperfect water suppression in the clinical data.

The CNN block was inspired by the AUTOMAP network [24], customized for one-dimensional data. The model comprises three fully connected layers with hyperbolic tangent activation, succeeded by two convolutional layers with ReLU activation [25], and a deconvolution layer. The training utilized mean-squared-error loss and the Adam optimizer [26], employing a batch size of 20, a learning rate of 5e^-6^, and 200 epochs. To leverage the distinct information each component of the spectrum provides, the real and imaginary components of the spectra were trained separately within the same model. During each epoch, the training set underwent processing through the model to generate frequency domain spectra. Subsequently, the mean squared error (MSE) was calculated, and the model’s weights were updated through backpropagation. This iterative process continued throughout the epochs, refining the model’s performance over time.


$$\text{MSE }=\frac{1}{N}\overset{N}{\underset{i=1}{\sum }}{\left(y_{i}-{\hat{y}}_{i}\right)}^{2}$$ (1)

The DENSENET block was inspired by DenseNet [27], tailored for one-dimensional time series data (Fig 1). DenseNet’s distinctive densely connected structure enables the training of deep models without excessive computational demands, minimizing overfitting. The model incorporates ReLU activation [25], and to adapt it for binary classification a sigmoid activation was appended at the model’s conclusion. Binary cross entropy (BCE) was used as the loss function for the backpropagation.


$$\text{BCE }=-\frac{1}{N}\overset{N}{\underset{i=1}{\sum }}y_{i}*\log\hat{y_{i}}+\left(1-y_{i}\right)*\log\left(1-\hat{y_{i}}\right)$$ (2)

### 2.3 Training

For training the model, the training dataset was divided into distinct training (80%) and validation datasets (20%). The test dataset for evaluating the performance of the classification model was reserved from one institute (40 patients with gliomas), and their data were not used in the training phases. The training dataset encompasses 20,000 MR spectra from healthy subjects and patients with glioma [22]. Each spectrum, consisting of real and imaginary components, has 1024 time points. To establish the ground truth in our study, we selected specific voxels based on detailed segmentation by two experienced neuroradiologists, each with over 10 years of experience, as documented in our previously published work [28]. For glioma voxels, we focused on voxels located within the tumor regions for each patient. To identify healthy voxels, we chose lateral voxels situated far from the tumor, specifically from the opposite brain hemisphere. To maintain the population balance between tumor and healthy MR spectra, we augmented the dataset with additional MR spectra derived from tumor regions. For the test dataset, we selected only a few voxel data points to ensure a representative and balanced evaluation. For the open-access data used for training, we selected voxels around the tumor regions in patients with gliomas and further augmented them for the “Glioma” group. For the “Healthy” group, we only used MR spectra from healthy subjects. This careful selection ensures that the healthy voxels are well-separated from the tumor region, reducing the likelihood of contamination from tumor-affected areas. This method of ground truth selection allows for a robust comparison between glioma and healthy brain tissue, providing a reliable basis for validating our model.

Training the classification model was done using binary cross entropy as the loss function, and the Adam optimizer for optimization [26]. A batch size of 200, a learning rate of 5e^-5^, and 100 epochs were employed during the training process. The model takes in raw time domain data, featuring separate channels for the real and imaginary components of the complex spectra. The final layer of the model is a fully connected layer with 1024×2 nodes, reconstructing the complex frequency spectra. The implementation utilized TensorFlow 2.5 [29] and Keras [30], and the training process occurred on a fourth-generation MacBook Pro with an Apple M1 chip, featuring an 8-core CPU, 8-core GPU, 16-core Neural Engine, and 16.0 GB RAM.

### 2.4 Evaluation

To evaluate the CNN block, where the true frequency domains were known, we processed the test dataset and calculated the MSE to assess performance and monitor for overfitting. For this block, the output variables used for both model training and testing were the entire frequency spectra. This approach allowed us to quantify the accuracy of spectral reconstruction by examining the overall discrepancy between the predicted and true spectra. The reconstruction was further evaluated by calculating the SNR, the peak signal-to-noise ratio (PSNR), the root mean squared error (RMSE), the structural similarity index measure (SSIM), and the correlation between the model outputs and the ground truth spectra. This thorough evaluation ensured that CNN’s predictions were closely aligned with the actual frequency domains, validating the model’s effectiveness in spectral data transformation.

In evaluating the classification performance of the model, we calculated Receiver Operating Characteristic (ROC) curves, a widely accepted metric in medical imaging and classification tasks. The ROC curves provided a comprehensive assessment of the model’s ability to discriminate between different classes, in the context of voxels containing brain tumor tissue (“Glioma”) versus healthy (non-tumor) tissue (“Healthy”) using MRSI raw data. The ROC analysis provides robustness to our findings and offers insights into the model’s sensitivity and specificity, which are crucial factors for clinical decision-making and diagnostic accuracy.

Confusion matrices were also calculated to further assess the performance of our model. The confusion matrix allowed for a detailed examination of the model’s classification outcomes, by dividing our results into true positives, true negatives, false positives, and false negatives. This comprehensive evaluation provides a nuanced understanding of the model’s precision, recall, and overall classification accuracy. The combined use of ROC curves and confusion matrices strengthens the robustness of our results, contributing to a thorough characterization of the model’s performance in the context of glioma vs. non-tumor tissue classification through *in vivo* MRSI data.

To investigate and visualize how our deep learning classification model distinguishes tumors from healthy non-tumor tissues, we utilized Grad-CAM (Gradient-weighted Class Activation Mapping) [31]. Grad-CAM helps us understand and interpret the model’s decisions by highlighting areas in the input data that significantly affect the model’s output. This involves performing a backward pass to compute the gradients of the target class score concerning the final convolutional layer’s feature maps.

## 3 Results

### 3.1 . Domain transformation

The domain transformation was conducted by the CNN block of the model. Synthetic, phantom, and *in vivo* MRSI data were employed to train and evaluate the model performance.

**Fig 2:**
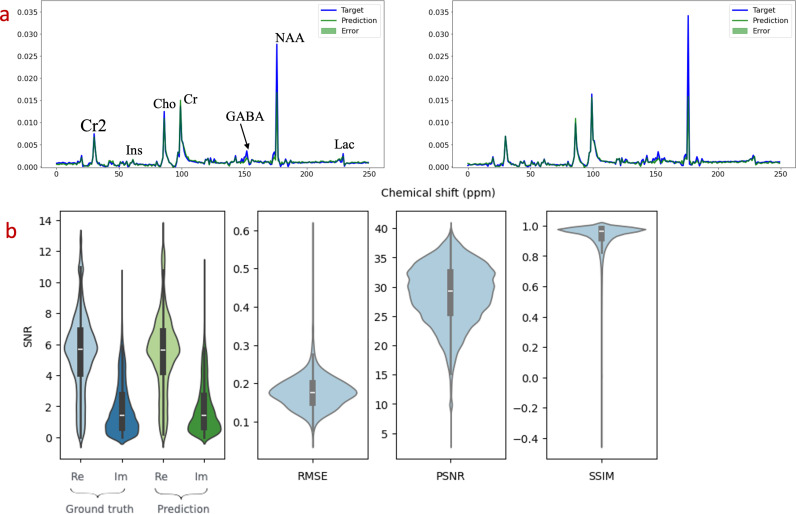
(a) Illustration from the comparison between MR spectra (blue spectra, acquired from the brain phantom) and the corresponding model predictions using the CNN block (green spectra). This visual representation provides a clear insight into the performance of the model in predicting the frequency domain spectra when compared to the actual MR spectra. (b) SNR comparison between the vivo MR spectra and the predicted spectra, and RMSE, PSNR, and SSIM test between generated MR spectra and in-vivo spectra. The model gives high similarities in these image-based assessments.

The trained model was evaluated using an unseen test dataset (greater than 10,000 MR spectra). The model demonstrated an excellent fit with the original MR spectra in the frequency domain, as indicated by the MSE (SD), ranging from 10^-6^ – 5.510^-4^, depicted in Fig 2a. Fig 2b shows a similar SNR distribution in the spectra between the model-generated spectra and the *in vivo* data, and the RMSE, PSNR, and SSIM metrics further prove the quality of the generated spectra with regard to the experimental spectra. The generated spectra have a mean SSIM of 0.92 ± 0.01.

### 3.2. Densely deep learning model classified MR spectra obtained from gliomas versus healthy voxels

**Fig 3:**
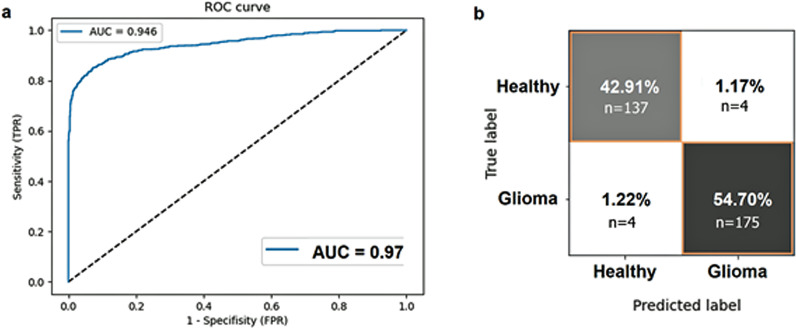
(a) Receiver-operator characteristic curve (ROC) with an Area Under the Curve (AUC) of 0.95 for classification of glioma vs healthy tissue. (b) The confusion matrix of the test set, reveals accurate classification with minimal errors. Specifically, there are few false positives (1.17% of the total, predicted glioma) and few false negatives (1.22% of the total, predicted healthy).

The ROC probability curve for the test dataset, depicted in Fig 3a, illustrates our model’s ability to balance sensitivity and specificity, emphasizing the discriminative power of the model in terms of the Area Under the Curve (AUC). AUC is a measure of how well two groups can be distinguished. The AUC of 0.95 was achieved for the commendable predictive power of our model in distinguishing glioma from healthy voxels. Next, we evaluated the model’s performance in classifying gliomas from healthy MR spectra using a confusion matrix. Our test dataset comprised a well-balanced representation of glioma and healthy cases to ensure a comprehensive assessment of the model’s classification accuracy. The confusion matrix, depicted in Fig 3b, provides four interpretations of (i) true positives (54.70%, dark gray box): the number of instances where the model correctly classified glioma voxels; false negatives (1.22%, white box): instances where the model incorrectly predicted healthy when the actual class was glioma; false positives (1.17%, white box): instances where the model incorrectly predicted glioma when the actual class was healthy; true negatives (42.91.8%, light gray box): The number of instances where the model correctly classified healthy cases. The overall sensitivity and specificity of the classification model were 97.8% and 97.3%, respectively.

**Fig 4:**
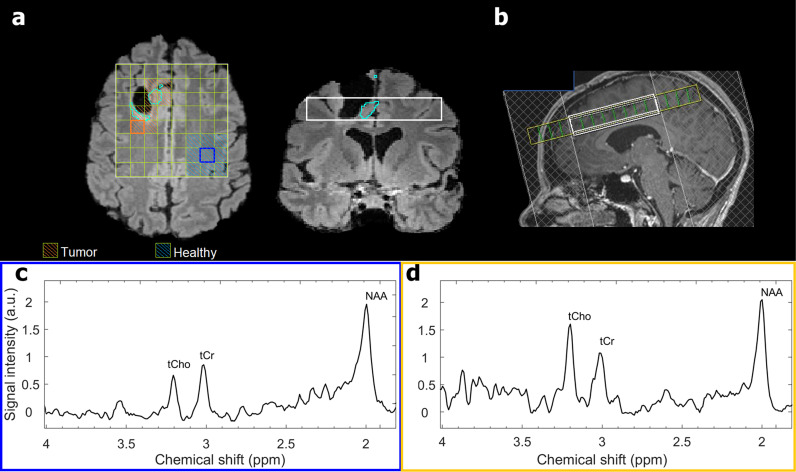
(a) Cross-sectional images in the axial and coronal planes generated after image processing; and (b) sagittal view, showing the MRSI grid positioning acquired using 3 Tesla 2D-PRESS-MRSI with a 16 × 16 matrix for a female patient with glioma (31 years old, CNS WHO grade 3 oligodendroglioma, frontal right, IDH1 mutated, recurrence) during scanning, depicting FLAIR (fluid-attenuated inversion recovery). In (a), we illustrate the 8×8 grid on the axial view within the shim box (white contour) and the selected voxels included in the test dataset for this subject (squares with oblique lines). Cyan-colored contours demonstrate the tumor-containing regions of recurrence verified and drawn by neuro-radiologists. Examples of MR spectra are shown from the highlighted voxels (tumor, orange; healthy, blue), showing an increased total choline (tCho) signal compared to total creatine (tCre) and N-acetyl aspartate (NAA) in the viable tumor area and adjacent tissues, while NAA and tCre demonstrate higher intensities compared to tCho in the adjacent healthy regions. (c) Illustrative MR spectra are presented for healthy tissue (from the same patient) and (d) adjacent to the site of tumor recurrence.

The proposed model, designed for spectral time series analysis exhibited promising outcomes. Trained on a diverse dataset encompassing synthetic spectroscopic data, phantom data, and real data from brain tumor patients, the model demonstrated its efficacy in discerning the presence of tumor lesions. The model’s success in domain transformation from raw data, as demonstrated in the synthetic MR spectra and phantom data experiments, laid the groundwork for its subsequent performance on *in vivo* data. The fine-tuning process further optimized the model’s weights, enhancing its adaptability to real-world clinical scenarios. The model could classify MR spectra from glioma tissue and distinguish them from those of healthy, non-tumor tissue (e.g., Fig 4 voxels marked with blue-colored oblique lines vs. those with orange-colored oblique lines).

### 3.3. Grad-CAM derived saliency maps depicted metabolite’s contribution in the classification task

Grad-CAM analysis was subsequently applied to visualize the decision-making process of the model during the training phase when classifying healthy spectra from glioma ones. The model demonstrated a particular interest in the area near the choline peak for glioma evaluation, with activation also observed around 2.25 ppm. Conversely, for assessing healthy spectra, a larger portion of the spectrum was activated, particularly focusing on areas around NAA.

## 4 Discussion

In this study, we proposed a supervised deep learning model for classifying unprocessed raw MR spectra of healthy tissues from those of glioma tissues. Direct use of time-domain spectra acquired from the scanner without preprocessing can improve accessibility and potentially bypass the need for additional preprocessing. Our findings highlight the significant potential of deep learning in the realm of MRSI for brain tumor classification. The demonstrated success of our model in distinguishing tumor tissue from healthy tissue based on raw MRSI data in the time domain underscores the promise of automated, data-driven approaches to enhance diagnosis and streamline the analysis pipeline.

Synthetic data can greatly benefit the research community and is arguably crucial for MR imaging and other imaging modalities, especially when access to clinical data is limited or group populations are imbalanced when examining diseases using deep learning. [32–34]. To improve our model’s training, we generated and used synthetic spectroscopy data. We simulated metabolite signals based on known chemical shift patterns, added realistic noise levels, and mimicked clinical data variations. This ensured that the synthetic spectra accurately reflected real MRS data, enhancing the model’s reliability and performance.

Machine learning models have shown considerable potential in classifying healthy tissues from glioma and other cancerous tissues. For example, using Raman spectroscopy on 63 biopsies from adult subjects undergoing surgery, Riva et al. [35] achieved a sensitivity of 83% using a supervised gradient boosting trees technique. In *in vivo* studies, deep learning models have emerged as valuable tools, addressing challenges in balancing sufficient SNR with higher spatial resolution in MRSI and other advanced MRI modalities. In a notable study by Hatami et al. [14], CNN outperformed standard quantification methods and simpler machine learning models, such as random forests. Lee and Kim [17] employed a CNN to predict spectra containing only metabolites based on noisy input MR spectra. This model can be viewed as a form of pseudo-fitting, where metabolite concentrations can be determined by line fitting on the predicted spectra or estimated using simplified methods. Gurbani et al. [16] implemented an autoencoder approach for spectral fitting, predicting parameters to capture metabolite peaks and employing wavelet reconstruction to generate a baseline. Additionally, the same group developed a model to identify and discard MR spectra of poor quality by recognizing common artifacts [15]. While this approach reduces the workload by removing anomalous spectra, the processing and analysis of acceptable spectra still require expert intervention. Our proposed model, however, classifies raw time-domain MR spectra, eliminating the need for spectral analysis and investigation in the frequency domain.

Koonjoo et al. [36] investigated the use of the AUTOMAP deep learning framework, designed specifically for transforming lower SNR images acquired at ultra-low-field. They successfully illustrated the utility of the AUTOMAP-based image reconstruction approach across diverse low-field MRI datasets obtained in the mT regime, demonstrating significant improvements in both SNR and image quality. In our study, we adapted AUTOMAP for one-dimensional time series MR data, incorporating a blend of low and high SNR MR spectra. However, during the initial experiments utilizing solely synthetic data, we observed a tendency for our training with the AUTOMAP-derived structure to overfit the data. To address this issue, we introduced more variation in augmentation and incorporated phantom MRS data into the training process. For the second block of the model, we used DenseNet architecture. Previous studies demonstrated that DenseNet yields more robust results and generalized well to unseen data [27,37–39]. While the DenseNet network effectively performed the classification, a comprehensive evaluation of the generated frequency domain spectra is crucial, necessitating a comparison to standard methods. By combining the AUTOMAP and DenseNet-derived architectures, we achieved more robust classification performance on unprocessed time-domain spectra, correctly predicting 95% of the total MR spectra in the test dataset, evaluated by the ROC metric to assess the quality of output. In future research, we aim to assess the model’s performance on other multi-center clinical MRSI data and include clinical and demographic data in the training phase. For such an experiment, we will need to acquire more *in vivo* subject data.

**Fig 5:**
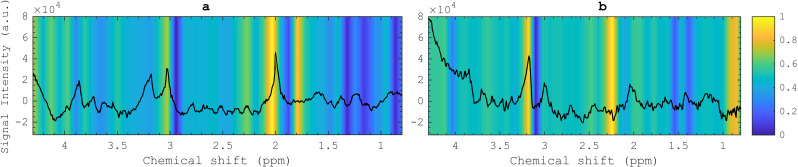
Illustration of the significant spectral regions contributing to the classification of MR spectra originated from glioma and healthy tissues. An example of a spectrum from each class is represented as a reference in solid black, while color-coded stripes indicate the regions of importance generated using the Grad-CAM method. These areas were identified using stability feature selection: (a) Healthy spectrum – the areas around the 1.8 ppm, 2.0 ppm: NAA and NAAG peaks, and 3.0 ppm: total creatine is the most active. (b) Glioma spectrum – the area around the 3.2 ppm: total choline peak and resonance region of 2.25 ppm: GABA, glutamine, glutamate, and 2-Hydrogyglutarate are among the highlighted regions.

Grad-CAM analysis identified an expanded portion of the spectrum in MR spectra classified as “Healthy,” with emphasis on regions surrounding NAA, a crucial metabolite that serves as a biomarker for neuronal density in brain tissues [4,40]. A reduction in NAA may suggest unhealthy brain tissue, neuron loss, or neuronal replacement by other cell types. In contrast, in glioma MR spectra, Grad-CAM highlighted the spectral regions near the choline peak (3.2 ppm) and around 2.25 ppm as key features for glioma classification (Fig 5). Previous studies have consistently shown elevated levels of choline-containing metabolites relative to NAA and Cr as reliable sensitive cancer biomarkers, often expressed as the Cho/Cr or Cho/NAA ratio [4,41–43]. These metabolite ratios are typically concentrated in tumor regions, providing valuable information for surgical planning and complementing other imaging techniques in precision medicine [43–45]. The region around 2.25 ppm, highlighted in Grad-CAM analysis, corresponds to critical metabolites such as glutamine, glutamate, and 2-hydroxyglutarate (2-HG), with the latter serving as a unique biomarker for identifying *Isocitrate dehydrogenase (IDH)* mutations and cancer cells [46]. The accumulation of 2-HG likely accounts for the regions prominence in classification, given that 52% of glioma patients in the test dataset harbored *IDH* mutations. As a metabolite uniquely associated with *IDH* mutations, 2-HG serves as a critical biomarker for identifying this mutation event in gliomas [47]. Testing for 2-HG levels can aid in the diagnosis of *IDH*-mutated tumors and inform treatment decisions. However, the clear detection of 2-HG, as well as its distinction from overlapping glutamine and glutamate signals, remains challenging with conventional spectral processing methods. Our AI-based model’s sensitivity to 2-HG highlights its holds promise and potential in distinguishing *IDH*-mutated gliomas from wild-type gliomas based on raw spectroscopic data, as well as differentiating grade 2 from grade 3 gliomas—both of which could have significant clinical implications. However, to fully harness this potential, further investigation is necessary, involving a more comprehensive spectroscopic dataset that includes diverse glioma subtypes, demographic variability, and data from multiple institutions to ensure broader validation.

There are several potential directions for future research, given that this study is limited to an input size of 1024 spectral points and a specific range of TEs. Future investigations could explore different representations of the input data, such as using multiple TE acquisitions and additional data points. Additionally, networks could be trained and evaluated on a variety of pulse sequences and timings to assess their generalizability. Importantly, due to the lack of *in vivo* MRSI data, we were unable to examine 3-dimensional MRSI or fully include 2D MRSI voxels. Our approach minimizes user interaction, reduces computation time, and ensures that clinically relevant data is highlighted for the classification of MR spectra. However, it is not yet fully automated. Additional steps are necessary to achieve fully automated raw MRSI data analysis. Altogether, providing a comprehensive platform capable of performing domain transformation, classification, and quantification of individual metabolites [48] could be of great clinical interest.

## 5. Conclusion and future perspectives

In conclusion, the demonstrated robustness in domain transformation, spanning from synthetic spectra to *in vivo* data and refined through the fine-tuning process, underscores the adaptability of our model to diverse datasets and real-world scenarios. The model’s efficacy in distinguishing glioma from healthy MR spectra further positions it as a valuable tool with potential clinical applications. The integration of MR spectroscopy with deep learning algorithms holds immense promise for improving the accuracy of *in vivo* investigation of lower-grade gliomas. As these techniques progress and gain broader acceptance in clinical settings, they have the potential to streamline the diagnostic and prognostic evaluation of glioma patients, providing valuable guidance for clinical decision-making.

However, further research is imperative to validate the performance of these integrated approaches across larger, multi-center datasets and to assess their impact on patient outcomes. Alternative data augmentation techniques, such as variation in echo-time and inclusion of 3D MRSI data could enhance model training and reduce the risk of overfitting. Future research should incorporate additional evaluation metrics, such as sensitivity to glioma subtypes. Standardizing data collection, evaluation criteria, and reporting guidelines will be crucial to ensure the generalizability and reliability of these methods in diverse clinical scenarios. This ongoing exploration and refinement are essential steps toward establishing the clinical utility of integrated MRSI and deep learning in the management of gliomas, particularly in examining and distinguishing lower-grade (grade 2 versus 3) gliomas in future applications.
